# Supplementary material for: Impact of Different Lignin Sources on Nitrogen−Doped Porous Carbon toward the Electrocatalytic Oxygen Reduction Reaction
Source: Int J Environ Res Public Health. 2023 Mar 1;20(5):4383. doi: 10.3390/ijerph20054383 (PMC10002350; doi:10.3390/ijerph20054383)
Supplement: Supplementary file 1 [file ijerph-20-04383-s001.zip › ijerph-2192950-supplementary.pdf]

Supporting information

1. Supplemental tables

Table S1. Comparison of ORR performance of different lignin catalysts

Precursor	E_{onset}(V)	$E_{1/2}$(V)	J_d(mA·cm^{-2})	Tafel(mV / decade)	Reference
Enzymolytic lignin	0.93	0.82	1.33		
Alkali lignin	0.96	0.84	2.03		
Dealkali lignin	0.79	0.6	1.38		
Lignin	0.892	0.792	6.9	43	[1]
Lignin	0.94	0.9	4.9	51	[2]
Bagasse lignin	0.92	0.86	4.67	71	[3]
Lignin extracted from beech wood	0.89	0.85	5.4		[4]
Alkali lignin	0.3	0.35	4.9		[5]
Lignosulfonate	0.8	0.75	2.9	38.5	[6]
Lignin	0.17		3.9		[7]
Alkali lignin	0.97	0.84	7.06		[8]
Pennisetum lignin	0.92	0.86	6	40.87	[9]
Alkali lignin	0.959	0.844	4.26		[10]
Alkali lignin	0.95	0.851	6.27	80.5	[11]

2. References

1. Shen, Y.; Li, Y.; Yang, G.; Zhang, Q.; Liang, H.; Peng, F., Lignin derived multi-doped (N, S, Cl) carbon materials as excellent electrocatalyst for oxygen reduction reaction in proton exchange membrane fuel cells. *Journal of Energy Chemistry* **2020,** *44*, 106-114.
2. Li, P.; Wang, H. L.; Fan, W. J.; Huang, M. H.; Shi, J.; Shi, Z. C.; Liu, S., Salt assisted fabrication of lignin-derived Fe, N, P, S codoped porous carbon as trifunctional catalyst for Zn-air batteries and water-splitting devices. *Chemical Engineering Journal* **2021,** *421*.
3. Shen, Y.; Peng, F.; Cao, Y.; Zuo, J.; Wang, H.; Yu, H., Preparation of nitrogen and sulfur co-doped ultrathin graphitic carbon via annealing bagasse lignin as potential electrocatalyst towards oxygen reduction reaction in alkaline and acid media. *Journal of Energy Chemistry* **2019,** *34*, 33-42.
4. Graglia, M.; Pampel, J.; Hantke, T.; Fellinger, T.-P.; Esposito, D., Nitro Lignin-Derived Nitrogen-Doped Carbon as an Efficient and Sustainable Electrocatalyst for Oxygen Reduction. *Acs Nano* **2016,** *10* (4), 4364-4371.
5. Rois, M. F.; Widiyastuti, W.; Setyawan, H.; Rahmatika, A. M.; Ogi, T., Preparation of activated carbon

from alkali lignin using novel one-step process for high electrochemical performance application. *ARABIAN JOURNAL OF CHEMISTRY* **2021,** *14* (6).

6. Zhang, M.; Song, Y.; Tao, H.; Yan, C.; Masa, J.; Liu, Y.; Shi, X.; Liu, S.; Zhang, X.; Sun, Z., Lignosulfonate biomass derived N and S co-doped porous carbon for efficient oxygen reduction reaction. *Sustainable Energy & Fuels* **2018,** *2* (8), 1820-1827.
7. Demir, M.; Farghaly, A. A.; Decuir, M. J.; Collinson, M. M.; Gupta, R. B., Supercapacitance and oxygen reduction characteristics of sulfur self-doped micro/mesoporous bio-carbon derived from lignin. *MATERIALS CHEMISTRY AND PHYSICS* **2018,** *216*, 508-516.
8. Zhang, X. L.; Yu, D. L.; Zhang, Y. Q.; Guo, W. H.; Ma, X. X.; He, X. Q., Nitrogen- and sulfur-doped carbon nanoplatelets via thermal annealing of alkaline lignin with urea as efficient electrocatalysts for oxygen reduction reaction. *RSC ADVANCES* **2016,** *6* (106), 104183-104192.
9. Li, C.; Wu, Y.; Fu, M.; Zhao, X.; Zhai, S.; Yan, Y.; Zhang, L.; Zhang, X., Preparation of Fe/N Double Doped Carbon Nanotubes from Lignin in Pennisetum as Oxygen Reduction Reaction Electrocatalysts for Zinc-Air Batteries. *ACS APPLIED ENERGY MATERIALS* **2022,** *5* (4), 4340-4350.
10. Wang, Y.; Gan, R.; Zhao, S.; Ma, W.; Zhang, X.; Song, Y.; Ma, C.; Shi, J., B, N, F tri-doped lignin-derived carbon nanofibers as an efficient metal-free bifunctional electrocatalyst for ORR and OER in rechargeable liquid/solid-state Zn-air batteries. *Applied Surface Science* **2022,** *598*, 153891.
11. Huang, B.; Jiang, J., N-doped carbon nanosheets derived from lignin as a novel bifunctional electrocatalyst for rechargeable zinc-air battery. *Diamond and Related Materials* **2022,** *128*, 109291
